# Seasonal Dynamics of Ant Community Structure in the Moroccan Argan Forest

**DOI:** 10.1673/031.012.9401

**Published:** 2012-08-12

**Authors:** Abderrahim El Keroumi, Khalid Naamani, Hassna Soummane, Abdallah Dahbi

**Affiliations:** ^1^ Laboratory of Biotechnologies-Biochemistry, Valorisation and Protection of Plants (2BV2P), Faculty of Sciences Semlalia, Cadi Ayyad University, Marrakesh, Morocco; ^2^ Equipe de recherche Environnement et santé (ENSA), Département des Sciences Naturelles, Faculté Polydisciplinaire, Université Cadi Ayyad Safi, Marocco

**Keywords:** diversity, formicidae, *Monomorium subopacum*, richness, *Tapinoma simrothi*

## Abstract

In this study we describe the structure and composition of ant communities in the endemic Moroccan Argan forest, using pitfall traps sampling technique throughout the four seasons between May 2006 and February 2007. The study focused on two distinct climatic habitats within the Essaouira Argan forest, a semi-continental site at Lahssinate, and a coastal site at Boutazarte. Thirteen different ant species were identified, belonging to seven genera. *Monomorium subopacum* Smith and *Tapinoma simrothi* Krausse-Heldrungen (Hymenoptera: Formicidae) were the most abundant and behaviorally dominant ant species in the arganeraie. In addition, more specimens were captured in the semi-continental site than in the coastal area. However, no significant difference was observed in species richness, evenness, or diversity between both sites. Composition and community structure showed clear seasonal dynamics. The number of species, their abundance, their diversity, and their evenness per Argan tree were significantly dissimilar among seasons. The richness (except between summer and autumn), and the abundance and the evenness of ant species among communities, showed a significant difference between the dry period (summer and spring) and the rainy period (winter and autumn). Higher abundance and richness values occurred in the dry period of the year. Ant species dominance and seasonal climatic variations in the arganeraie might be among the main factors affecting the composition, structure, and foraging activity of ant communities. This study, together with recent findings on ant predation behavior below Argan trees, highlights the promising use of dominant ant species as potential agents of Mediterranean fruit fly bio-control in the Argan forest and surrounding ecosystems.

## Introduction

Despite the crucial importance of ants and their position as keystone species in most terrestrial ecosystems, their role, ecology, and diversity dynamic patterns are not fully understood, a fact that greatly undermines their potential usefulness in agro-forest conservation and pest management programs.

Ants are important components of the majority of terrestrial ecosystems in terms of biomass and diversity, playing a crucial role in their function (Hölldobler and Wilson 1990). They are major arthropod predators that regulate many insect populations (Jeanne 1979; Novotny et al. 1999; Cerdá and Dejean 2011), especially those found in or on the ground, such as soil pupating Diptera and Lepidoptera. Given their stabilizing and/or regulating effect on insect populations, ant and plant interactions are widespread in plant communities, and often involve a protective role against potential pests and herbivores (Schupp and Feener 1991). Ants were the first insects to be used as biological control agents, and are still in use today for this purpose. Recently, several species have been incorporated into various programs of integrated pest management (IPM), and conservation plans in various ecosystems (Way and Khoo 1992; Perfecto and Castiñeiras 1998; Eubanks 2001). In fact, several ant species, such as some *Oecophylla*, have been efficiently used as biocontrol agents against phytophagous insects in natural and agro-ecosystems (Majer 1976; Folgarait 1998; Kenne et al. 1999). Thanks to their highly aggressive and territorial behavior, they have been successfully deployed to manage major insect pests, including fruit flies, in a broad range of perennial tree-crop agro-ecosystems, including cashew, citrus, cocoa, coconut, and mango (Peng and Christian 2005; Van Mele 2008). Other ant species have also been reported as contributing to pest control in diverse perennial crops (Torres 1982; Majer 1986; Way and Khoo 1992; Perfecto and Castineiras 1998; Philpott and Armbrecht 2006).

Understanding the factors that characterize species' communities in their natural habitats is the basic step in the use of natural enemy as biological pest management projects. In ant communities, a plethora of abiotic and biotic complex factors affect the specific interactions, composition structure, and species diversity dynamic within ecosystems (Parr and Gibb 2010; Philpott et al. 2010), the relative importance of each one depending on the temporal and spatial scale (Levin 1992; Andersen 1995). Ant activity follows seasonal cycles that are generally correlated with physical changes in the environment, those changes mainly being temperature (Bollazzi and Roces 2002; Holway et al. 2002b) and food supply cycling (Steinberger et al. 1992). Andersen (1983) showed a pronounced seasonality in abundance, diversity, and species composition in a semi-arid Australian ant community. Recently, Basu (2008) analyzed seasonal and spatial patterns in ground foraging ants, and observed marked seasonal fluctuations. In the Spanish Mediterranean ant communities, Retana and Cerdá (1998) reported that seasonal patterns in community structure depended on temperature fluctuation. Other factors, such as vegetation, biotic interactions, and habitat disturbance regime (Andersen 1995) may influence both spatial and temporal foraging activity patterns in ant assemblage, especially within areas under intensive anthropogenic activity such as the Mediterranean region (Collins and Smith 2006). Recently, their particular response to biotic and physical disturbance has made ants a spectacular monitoring taxon in agro-ecosystems. For instance, the impact of the disturbance caused by agricultural practices on ant diversity and their protective function against pests was examined in a variety of perennials such as cocoa, banana, and coffee (Perfecto and Vandermeer 1996; Philpott and Armbrecht 2006). Analyzing and understanding the factors that organize the diversity and dynamics of ant communities' structure is critical to preserving ecosystem functions and services provided by ants in the Argan forest.

The Argan tree (*Argania spinosa* (L.) Skeels) is an endemic and emblematic forest tree of southwestern Morocco, belonging to the tropical Family Sapotaceae. It constitutes a typical agro-forestry ecosystem, and is likely to have positive repercussions on the conservation of biological diversity and ecological richness, as well as on the interaction stability of the sympatric species of fauna and flora. However, the Argan forest (arganeraie), which covers more than 820,300 hectare in southern Morocco (Travis et al. 2002), is an enormous reservoir of proliferation and dissemination of *Ceratitis capitata* Wiedemann (Diptera: Tephritidae) (Sacantanis 1957), the harmful polyphagous and cosmopolitan Mediterranean fruit fly. This pest induces serious economic losses in arganeraie production, as well as in fruiting plants in neighbouring orchards. Intriguingly, despite their potential usefulness in the control of this pest, no complete data related to the Argan forest myrmecofauna are available to date. Recent research in the Moroccan Argan forest highlighted the importance of ant predation on the Medfly population (El Keroumi et al. 2010). In fact, four ant species predated about 20% of pest larvae under Argan trees while searching for a pupation site on the ground. In this earlier work, it was suggested that ant predation might be useful as a biological control element in the Argan forest, and in the neighbouring Citrus orchards. But for this purpose, wider investigation focusing on ecological parameters, such as species inventory, abundance, diversity, and ant community dynamics, is needed.

**Figure 1.**
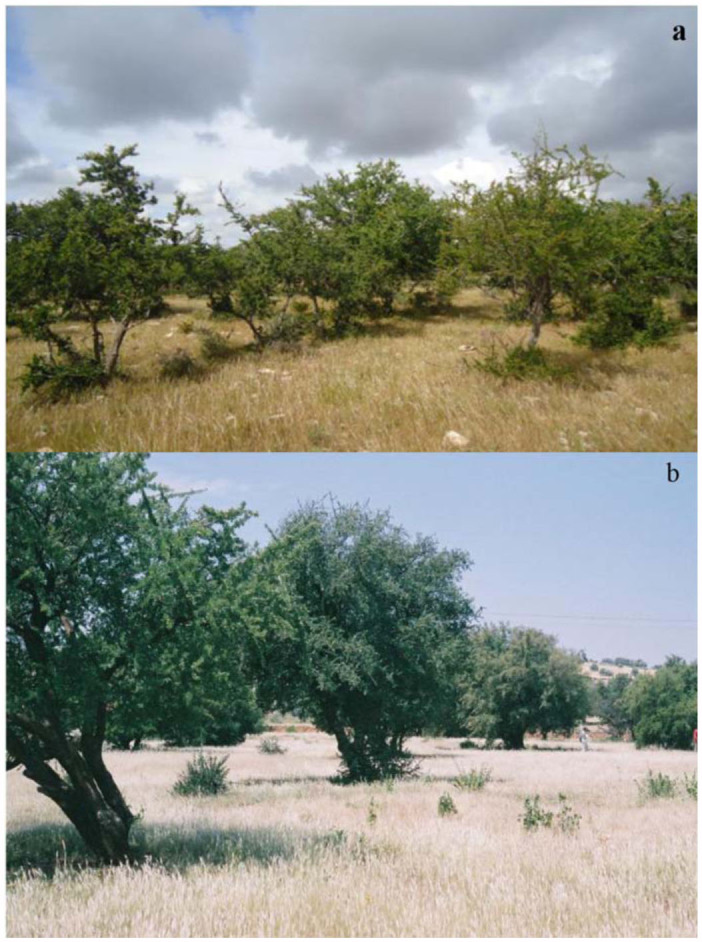
Photographs showing the physiognomy of the two arganeraie study sites. a) Boutazarte (coastal site), and b) Lahssinate (semi-continental site). High quality figures are available online.

The objectives of the present study are to characterize arganeraie ant communities, and to survey the seasonal variation of these communities. Results obtained from this work might be useful in assessing the spatial and temporal variation of the foraging activity of ant communities. It will also provide an insight into the importance of ant species as biocontrol agents.

## Materials and Methods

### Study sites

The study was conducted in Essaouira region (western Morocco), on the northern side of the western High Atlas. This region had an arid to semi-arid Mediterranean climate, having a cold winter, a rainy spring and autumn, and a dry, hot summer. Sampling was carried out at two sites, about 50 km apart, in Boutazarte (the coastal site) and Lahssinate (the semicontinental site). Physiognomy and other characteristics of each site are given in Table 1 and Figure 1.

In Boutazarte, the vegetation cover was dominated by herbs and perennial herbaceous plants (*Chamaerops humilis* L., *Zizyphus lotus* L., and *Asphodelus albus* L.), occupying almost 55 % of the total area. The soil litter ranged from 40 to 80 mm, and varies widely below trees. The Argan trees in this coastal area constituted a relatively dense matorral of medium height; indeed, the Argan tree prospers in the internal domain (Quezel et al. 2003). The ground was sandy with stones, and anthropogenic activity was relatively low. In fact, this site was protected against grazing (Figure 1a).

Lahssinate was an open arganeraie orchard characterized by more sparse vegetation with wheat (*Triticum spp. L.*), barley (*Hordeum vulgare* L.), and seasonal cultures including Argan trees and other cultivated fruit-bearing trees (*Olea europaea* L., *Vitis vinifiera* L., *Ficus carica* L., and *Opuntia ficus indica* Mill.). The arganeraie in this zone constituted a degraded matorral with more open areas. Trees were spaced 5 to 50 meters apart, and had an average height of 9 meters (Figure 1b). This site was characterized by a loamy, stony ground, and significant human activity, as it is commonly exposed to grazing.

The two sites differed slightly in terms of their climate. Summers were dry and hot, with temperatures reaching 45° C and 37° C at Lahssinate and Boutazarte, respectively, in July through August. Winters were rainy, and the maximum monthly average precipitations were registered between November and February. Relative humidity was very characteristic of the region of Essaouira, which is always above 75% characterizing the littoral band (Delannoy 1980; Bellichi 1998).

### Ant sampling

Ant sampling was carried out with pitfall traps. Pitfall traps consisted of 7 cm diameter, 9.5 cm high plastic recipients, partially filled with soapy water. In each site, 10 sampling units (argan trees) were chosen, and a group of seven pitfall traps per tree were installed at equal distances from each other, within a 6 m circle around the trunk of the sampling tree, and with one trap placed in the center near the trunk. A total of 70 pitfall traps were set at every site (10 trees x 7 pitfalls) between May 2006 and February 2007 (sampling dates: 14 May 2006, 30 July 2006, 21 November 2006, 15 February 2007). Pitfall traps were left for 24 hours before they were collected. All the biological material collected in the seven pitfall traps of an argan tree was pooled (sampling unit was the 7-pitfall group). In the laboratory, ant specimens were separated from other collected fauna, and conserved in ethanol (70%). Ant species identification was carried out by Professor Henri Cagniant (Toulouse, France) and Professor Jacques H. Delabie (Itabuna, Brazil).

For the analysis of the community, the following parameters were estimated: Species richness (S), total number of species recorded at a given site during a sampling period around an argan tree; total ant abundance (Ntot), total number of individual ants collected per tree during a sampling period; Shannon's index of diversity (H), it was estimated as H = -σ (pi log pi), where pi is the proportion of individuals of the *i*th species (pi = ni / Ntot; ni: number of individuals of the *i*th species; Ntot: total number of individuals of all species) in traps during the period of time considered; Pielou's evenness index (J), the relative distribution of individuals with which each species is represent is represented in sample, it is calculated as J = H / H max (the value of J varies between 0 (a single species dominates) and 1 (all species are equally abundant)), and Hmax is the maximum possible value of H, and is equivalent to lnS—thus E = H lnS.

### Resampling procedure

To estimate the number of total expected species in each sampled community, a sample-based randomization procedure from EstimateS (Colwell 2006) was used. Within each sample for every site and season, sample order was randomized 50 times, and the mean and SD of richness (S) were computed for the 10 trees considered as sample units in each locality. Chao1 estimator of species richness was computed for total expected species per sample, and represents an estimator of the true number of species in a given sample, based on the number of rare species within each community (Colwell and Coddington 1994). The Chao1 bias correction was used for the spring and winter samples at the Lahssinate site, and for spring and summer samples at the Boutazarte site. For the other samples, data gave a Chao estimated coefficient of variation for abundance distribution > 0.5 and, in this case, the procedure recommended to recompute Chao1 as the classic instead of the bias-corrected option (Colwell 2006).

To estimate ant richness and diversity in each season at the coastal and semi-continental sites, species accumulation curves were computed using EstimateS (Colwell 2006). For each sampling day, sample-based rarefaction curves were computed from empirical data of the 10 trees at each site. The expected richness function Mao Tau and 95% CI curve, and the Shannon diversity index means (and SD), were estimated from 50 randomizations of sample order from EstimateS (Colwell 2006). This method illustrates the rate at which new species are added to the inventory within a community. As the number of samples increases, an increasing number of species are sampled, reaching a plateau (Magurran 2004).

### Data analysis

Statistical analyses were performed using the Statistica Statistical Package, Version 6.0 (StatSoft 2004). Data were evaluated for normality and homogeneity of variances with Kolmogorov- Smirnov test. When necessary, the transformation Log (X + 1) was used to normalize data (Zar 1998). For each sample (tree), average values of richness (S), ant abundance (Ntot), diversity (H), and evenness index (J) were calculated. Spearman's rank correlation coefficients were then denoted to compare relationship dependence between community parameters among all samples. The total number of individuals captured per tree at different sites and seasons was compared by a one-way repeated measures ANOVA. Post-hoc test pair-wise comparisons of abundance, richness, diversity, and evenness were done using the Tukey's HSD between the sampling seasons of the year.

For the ant species composition, the multivariate analysis was performed using the program Past (Hammer et al. 2001), using the binary matrix (ant species absence or presence). To establish whether or not there were significant differences in species composition between sites and sampling seasons, through the comparison of the differences among the average rank similarities between samples, the one-way ANOSIM analysis of similarity was used by 10,000 permutations to compute signification. R statistics is the measure of dissimilarity between sites and season samples. Values of R that are close to zero indicate low dissimilarity, while values of R that are closer to 1 indicate high dissimilarity (Clarke and Green 1988). For each R-value. a corresponding p- value gives the significant difference at p < 0.05. All analyses of dissimilarity were done with the Bray-Curtis index, because it is the most appropriate for multivariate statistics, and is less affected by the number of rare species in the samples (Krebs 1989). The goodness-of-fit was verified by the stress index produced in NMDS analysis (Clarke 1993).

The similarity percentage (SIMPER) analysis was performed to determine which species were good discriminators of the differences in composition among sites (Clarke 1993). The SIMPER analysis gives the percentage of dissimilarity between the sampled sites, presenting the percentage of contribution of each species to this dissimilarity.

## Results

### Comparison of ant communities

In Essaouira Argan forest, in the coastal and semi-continental zones, a total of 12,514 ants were captured with pitfall traps during the four sampling periods, between May 2006 and February 2007 (see Appendix 1). On average, 138.55 ± 179.61 (mean ± SD) ant workers were caught per Argan tree. 73.33% of the individuals were captured at the semicontinental site, and 26.66% at the coastal site. Most ant species were more abundant at the semi-continental than at the coastal site, with the exceptions of *Cataglyphis albicans voucheri* (40 vs. 200 catches) and *Camponotus erigens* (6 vs. 15 catches), which showed higher abundance at the coastal site. When all samples were considered together, more ants (irrespective of the species) were captured per Argan tree at Lahssinate (mean ± SD = 139.80 ± 186.95, N = 40) than at the Boutazarte site (mean ± SD = 80.15 ± 103.66, N = 40, one way ANOVA, F1, 78 = 18.22, p < 0.001). However, mean diversity, mean ant richness, and mean evenness showed no significant difference between the two sites (Table 2). *Monomorium subopacum* and *Tapinoma simrothi* were the most abundant species, and represented 54.34% and 37.06%, respectively, of all captures.

The collected specimens belonged to seven genera. *Camponotus* (3), *Cataglyphis* (3), and *Messor* (3) were the most speciose genus, while *Tapinoma*, *Monomorium* and *Crematogaster* were represented with only one species for each genus (Table 3, Appendix 1). Spearman's rank correlation coefficients showed significant relationship between the richness and the total ant abundance per Argan tree (r_s_ = 0.62, p < 0.001, N = 80), and the richness and the Shannon diversity index (r_s_= 0 .56, p < 0.01, N = 80). However, a negative significant relationship was observed between richness and the evenness index per Argan tree (r_s_ = 0.58, p < 0.01, N = 80).

Ant species' composition under Argan trees presented low dissimilarity between the coastal and semi-continental site of study (one way ANOSIM, R = 0.11; p < 0.001).

Ant species' percentage contribution in the observed dissimilarity between communities in the four seasons was indicated by the SIMPER (Similarity Percentage). The species *M. subopacum*, *T. simrothi*, and *Aphaenogaster senilis* accounted for more than 67% of the dissimilarity between both ant communities (Table 3), especially based on species abundance variability between the two sites.

### Seasonality in ant communities

**Figure 2.**
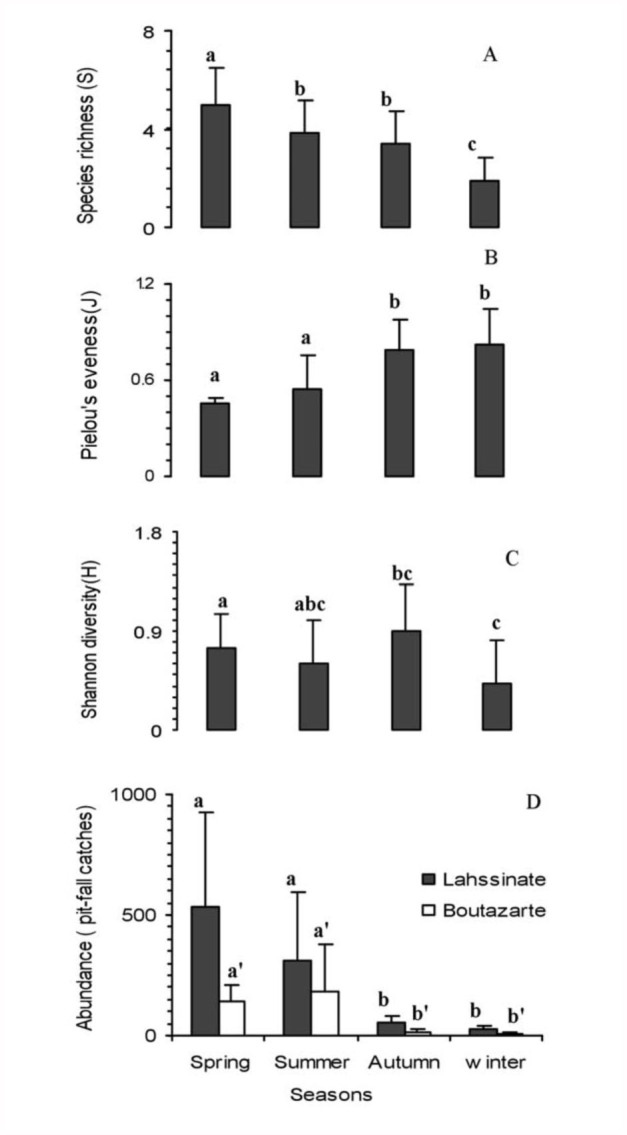
A) Ant species richness (S); B) Pielou's evenness index (J); C) Shannon's diversity index (H); and D) worker abundance at pitfall traps (mean values ± SE) during the four sampling periods. For all variables except abundance, there were no significant differences between sites and data were therefore regrouped. Different letters denote significant differences with Post-hoc test at P < 0.05. High quality figures are available online.

Considering all samples together, the one-way repeated measures ANOVA showed that ant communities in the Essaouira Arganaraie presented significant differences among seasons in mean richness (F_3,76_ = 22.74; P < 0.001), abundance (F_3,76_ = 17.47; P < 0.001), diversity (F_3,76_ = 18.78; P < 0.001), and evenness (F_3,76_ = 15.05; P < 0.001). Tukey's HSD Post-hoc test pairwise comparison of abundance (Figure 2D), richness (except between summer and autumn for the richness) (Figure 2A), and evenness (Figure 2B) showed a significant effect between seasons of the dry period (summer and spring) and the rainy period (winter and autumn). Higher total abundance values were observed in spring at Lahssinate, and in the summer at Boutazarte, but the lowest values were observed in the winter and autumn at both sites, with 51% of the total annual collection found in spring (40.8% in Lahssinate, and 10.2% in Boutazarte), 41% in summer (24.9% in Lahssinate, and 16.1% in Boutazarte), 5.1% in autumn (4% in Lahssinate, and 1.58% in Boutazarte), and only 2.42% in winter (1.8% in Lahssinate, and 0.62% in Boutazarete) (Figure 2D).

**Figure 3.**
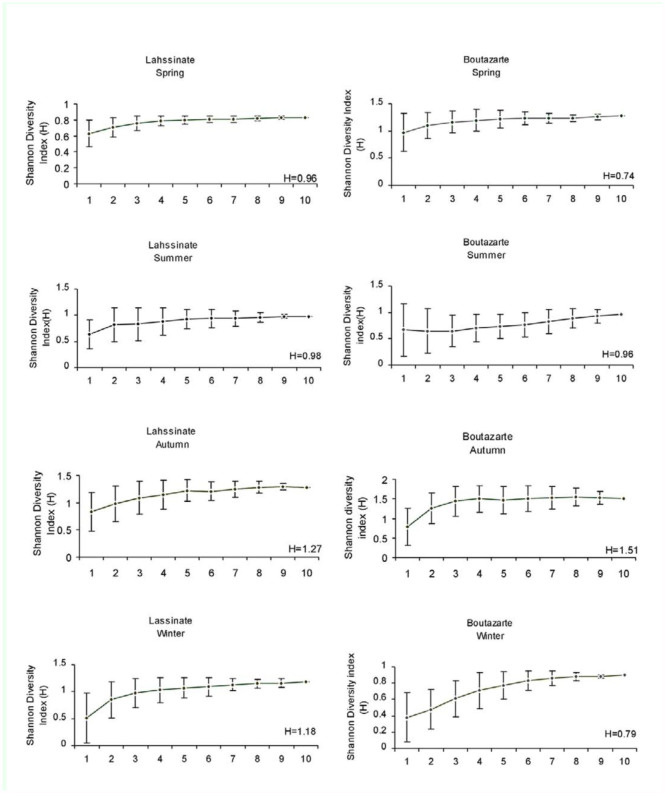
Comparison of ant diversity curves of the semi-continental (Lahssinate) and coastal (Boutazarte) sites in each sampling season. Values are the estimated Shannon diversity index mean (error bars: SD) from 50 randomizations of sampling order (from only one to ten sampling trees). Observed Shannon's diversity (H) is given for each sample season at the two sites. High quality figures are available online.

The highest richness mean values were observed in spring and summer, while the lowest values were in winter and autumn at both sites (Figure 2A). In spring, only two species were absent in the Lahssinate and Boutazarte samples (*Camponotus erigens* and *Aphaenogaster theryi*) among all ant species collected. In summer, two species of the total ant community were absent in the Lahssinate sample (*Messor capitatus* and *M. voucheri*), while four were absent in Boutazarte (*Crematogaster scutellaris algerica* and the three *Messor* species). In autumn, two species (*C. vaucheri* and *C. scutellaris algerica*) were absent in Lahssinate, and three species (*M. capitatus*, *M. vaucheri*, and *A. spp*. *Senilis*) in Boutazarte. During the winter, four species were present in the Boutazarte samples (*M. subopacum, T. simrothi, A. spp. senilis, and Camponotus lateralis*), and five species were collected in Lahssinate (*M. subopacum, T. simrothi, A. spp. senilis, M. vaucheri, and A. theryi*) (Figure 3). *M. subopacum* was the only species collected at all seasons in every unit sample (Appendix 1).

The highest values of Pielou's evenness index were observed in winter and autumn, but the lowest were found in summer and spring (Figure 2B). The Shannon's diversity index pairwise comparisons between the season samples showed that there was no significant difference, except between winter and spring (Figure 2C). Except for the mean number of ants collected per tree (F_3,76_ = 4.80, p < 0.004), no significant interacting effect was found between community parameters between the two sites in the four seasons sampled (Table 2). Ant species community structure differed between the hot and dry (summer and spring), and the wet and cold seasons (winter and autumn) (general ANOSIM, R = 0.3137, p < 0.0001).

**Figure 4.**
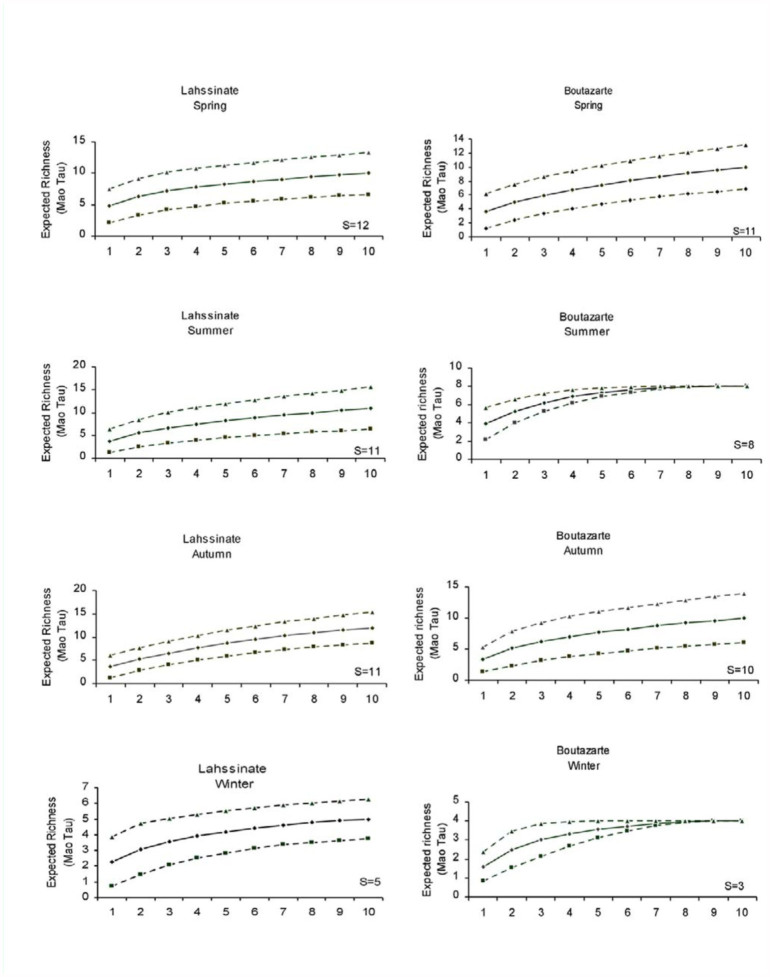
Sample-based rarefaction curves of the semi-continental (Lahssinate) and coastal (Boutazarte) sites and in each sampling season (sampling order was randomized 50 times. The values are the expected ant richness function Mao Tau and their 95% Cl. Observant ant species richness (S) is given for each sampling season at the considered sites. High quality figures are available online.

A low dissimilarity was found among the samples taken during the summer and spring (hot and dry) periods (R1 = 0.06488), and also for those taken during the winter and autumn (cold and rainy) periods, when samples were compared (R2 = 0.04791). By contrast, samples taken in winter and autumn showed a significant dissimilarity in ants' composition when compared with those taken in the summer and spring (Table 4).

Table 3 shows the ant species contribution percentage in the dissimilarities between ant species composition under Argan trees determined by SIMPER at different sampling seasons during the year. *M. subopacum, T. simrothi*, and *C. albicans vaucheri* may account for more than 68% of the seasonal ant community dissimilarities. By contrast, samples conducted in the autumn and winter seasons have shown significant dissimilarity in ants' composition when compared with those taken in the summer and spring (Table 4). This finding may underline the ecological importance of these species inhabiting the ground below Argan trees in the coastal and semi-continental zones of Essaouira Argan forest.

Because GLM do not remove the effects of abundance, a rarefaction approach was also used to compare communities at both sites between sampling seasons. Diversity curves from 50 randomizations at coastal and semicontinental sites are shown in Figure 3.The values of ant diversity in the coastal and semicontinental sites did not show any regular seasonal pattern for all samples. It is higher in winter and spring at the semi-continental site, but higher at the coastal site in the autumn. The lowest values of ant diversity were observed in spring at the semi-continental site, as well as at the coastal site. The highest values were observed in autumn, when almost equal diversity was found at the two sites in summer (Figure 3).

Figure 4 shows the expected richness curves of samples in different seasons from 50 randomizations of sample order. All the samples appear to be suitably carried out because, in general, the elevation of the curves tends to decrease, forming a plateau, as the number of new species decreases with the increasing number of trees taken as sampling units for almost all samples. In winter and summer samples at the Boutazarte site, the expected richness values (Mao Tau function) from resampling were similar to the observed richness (S), but observed values were slightly lower than the values expected in all other seasons in both sampling sites (Figure 4). This result is likely due to the effectiveness of the method used for sampling ants under Argan trees.

## Discussion

The species richness of ant communities in the Argan forest was relatively poor (13 species). This is most likely not due to the sampling methodology used in this study, since the estimated accumulation curves of ant species in both studied sites reached a plateau. However, two major reasons may account for the low species richness found within the Moroccan Argan forest. First, samples used in this study involved only the species living and/or foraging below Argan trees. Second, biodiversity in the Mediterranean ecosystems (such as Argan forests) is usually negatively affected by intense anthropogenic disturbances (Sala et al. 2000). Interestingly, others have also reported similar numbers of ant species in Mediterranean or semi-arid ecosystems. For instance, 14 ant species in Citrus orchards in Spain were reported (Cerdá et al. 2009, as well as between 6 and 15 ant species in other Spanish natural habitats (Retana and Cerdá 2000). Furthermore, a similar number was also found by Castracani et al. (2010) in some Italian habitats within the Castelporziano Natural Reserve area (13 species in a wet grassland, 14 species in a pine forest, and 13 species in a mixed oak forest). Heatwole and Muir (1989) found between 12 and 14 different species in the Tunisian steppes, and recently, Paknia and Pfeiffer (2011) found between 7 and 15 species in the Iranian arid steppes and deserts.

Among the Argan myrmecofauna, two ant species, *Monomorium subopacum* and *Tapinoma simrothi*, predominated, making up 92% of the total catches. Their relatively high abundance, combined with their true omnivorous regime (Delabie et al. 2000), may explain their ecological dominance, as occurs in other species of these genera (Aman et al. 2011; Blight et al. 2010; Cerdá et al. 2012; van Oudenhove et al. 2011). This also highlights their potential function as effective bio-control agents against pests within the Argan ecosystem. The activity of dominant species frequently reduces the foraging success of sympatric subordinates. This in turn affects species structure by contributing to the elimination of competitive species. This is the case in the South African savannah (Parr 2008), and in Spanish Mediterranean shrublands and forests. In these areas, dominant species are more abundant, and competitive forces appear to be the major mechanism organizing the foraging activity of each species (Retana and Cerdá 2000). The arganeraie situation is similar to that of other Mediterranean forests, where ant communities have a competitive structure based on dominant species. In fact, under Argan trees, *M. subopacum* and *T. simrothi* species display a substantial predatory activity on *C. capitata* larvae. All the other sympatric ant species do not interfere significantly with the population dynamics of the Medfly (El Keroumi et al. 2010). This observation seems to agree with the conception of Hölldobler and Wilson (1990), who proposed the ‘dominance-impoverishment’ rule, which states that a negative relationship exists between species richness and the degree of dominance in ant communities. Therefore, the fewer the ant species in a local community, the more likely the community is to be dominated by only one or a few dominant species. An impoverishment situation may occur within the Argan forest ant community, where the numerical dominance of *M. subopacum* species among Argan myrmecofauna seems closely related to its success in the predation of Medfly larvae. However, additional research on the interspecific competition in the Argan forest will be of great importance to confirm these suggestions.

### Effect of habitat on ant community structure

It was hypothesized that the Boutazarte site, which is directly exposed to the oceanic influences with a relatively high vegetation density and complexity, would create a wider potential diversity of available ecological niches. However, the findings were not consistent with this hypothesis, since ant species richness and diversity were, in general, not significantly different between both studied sites. This could be explained by the intrinsic needs of resources and biotope condition patterns required by each ant species (Hölldobler and Wilson 1990), and by the fact that a more structured and complex habitat does not necessarily provide the best environmental conditions for all groups of organisms. Even a reverse situation can sometimes be observed, as suggested by Retana and Cerdá (2000). According to Ricklefs and Schluter (1993), another hypothesis could be considered for the current study. At relatively smaller spatial scales (50 km distant sites), the effect of historical process differences in habitat structure could be even smaller to induce major variability in faunal diversity. However, in terms of total abundance, ant catches were more frequent at the semi-continental site compared to the coastal site (73.33% of the total annual collection vs. 26.67%). Only some rare species were very abundant at the littoral site, contradicting the general trend observed in these ant communities. Various combinations of ecological and climatic characteristics might generate a complex and heterogeneous mosaic of habitat patterns that affect resource availability and, as a consequence, the species community structure. At the Boutazarte site, habitat disturbance and direct exposure to the Atlantic Ocean, with a high relative air humidity always exceeding 75% (Delannoy 1980), could explain the reduction of ant abundance in the littoral band. Interestingly, the impact of ant abundance on the predatory rate on the larvae of the Medfly remained similar between the two sites (El Keroumi et al. 2010).

### Effect of seasonality on ant community structure

In this study, the phenological variation in community structure and composition is much higher than the variation between the two sampling sites, despite their specific disturbance regime. This result illustrates the importance of seasonality on the structure of Argan forest communities. The finding is comparable with those reported by Izhaki et al. (2003) in the longleaf pine forest in Florida, showing that the effects of natural seasonal cycles on ant community composition were more dominant than those of fire disturbance. Factors such as temperature and water availability in the environment are central factors regulating ant activity, because these affect overall plant biomass. The biomass affects ant activity because it defines the physiological needs and limits the activity of species, therefore acting on the temporal organization of community structure (Andersen 1983).

In the Argan forest, some ant ecological parameters (total abundance, richness (except between summer and autumn), and evenness) showed a marked seasonality. However, continentality had no significant effect on the total richness. At both sites, 12 ant species were active in summer, while only five species were active in winter. The variability in the foraging activity of ant community structure occurs in response to the marked climatic fluctuations between seasons that characterize Mediterranean ecosystems (cold winter, cool and rainy spring, dry and hot summer). The highest abundance values were observed in spring at Lahssinate, and in summer at Boutazarte, while the lowest values were noted in the winter at both sites. Indeed, higher temperatures in summer in the semicontinental zone, along with more open habitats in the Argan forest due to spare vegetation, could decrease the ant activity compared to the coastal site. These seasonal fluctuations are in accordance with those reported in other studies. In Australia, Andersen (1995) highlighted the involvement of temperature as the main abiotic stress factor regulating ant community, while in the Mediterranean ant communities, Cros et al. (1997) showed that seasonal ant activity patterns followed temperature fluctuations. The peak of ant foraging activity below Argan trees was observed in spring and summer, which coincides with the ripening period of Argan fruits, and thus maximizes the availability of *C. capitata* larvae when falling from fruit to the ground to find suitable pupating sites.

*M. subopacum* was the most abundant species, and it was characterized as the most efficient predator on the Medfly larvae (El Keroumi et al. 2010). The important ant foraging activity in the dry period, in particular that of *M. subopacum*, seems to be correlated with resource availability, in particular the abundance of Medfly larvae. These findings may explain the ecological dominance of *M. subopacum*, and its possible role in biodiversity conservation and bio-control projects in the Argan forest and adjacent agricultural orchards. In the Argan forest, beyond the clear seasonal fluctuations, ant community structure might be affected significantly by other macro and micro-scale factors, including biotic and abiotic disturbance, microhabitat effects, interspecific competition, and agricultural management. The study of various aspects of these combinations of factors could contribute even more to understand the role and ecological significance of ant species in the Argan forest.

**Table 1.**
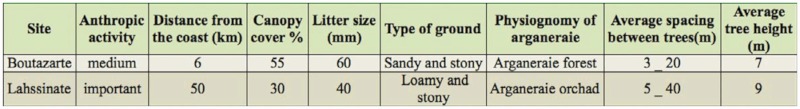
Main Characteristics of the study sites, Boutazarte and Lahssinate, in the Essaouira arganeraies.

**Table 2.**
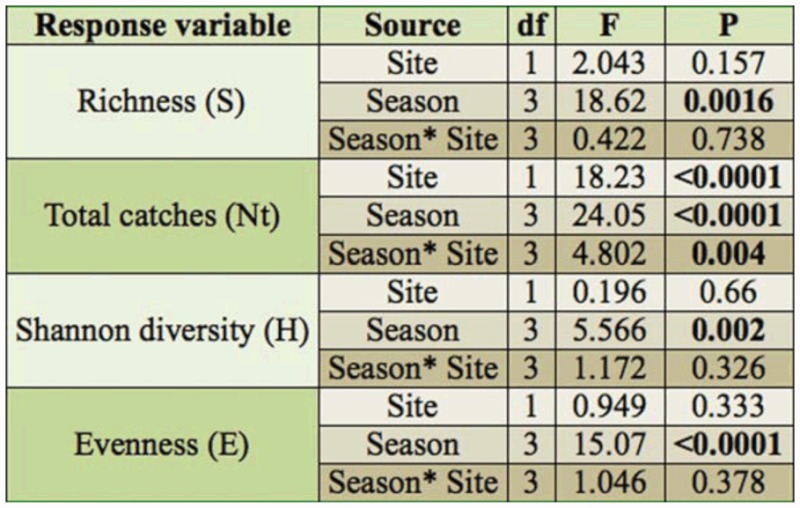
Repeated-measure ANOVA analysis testing effects of sampling site, season, and their interaction effects on ant species richness, total individual catches (Ntot), Shannon diversity (H), and Evenness (E) in the Argan forest.

**Table 3.**
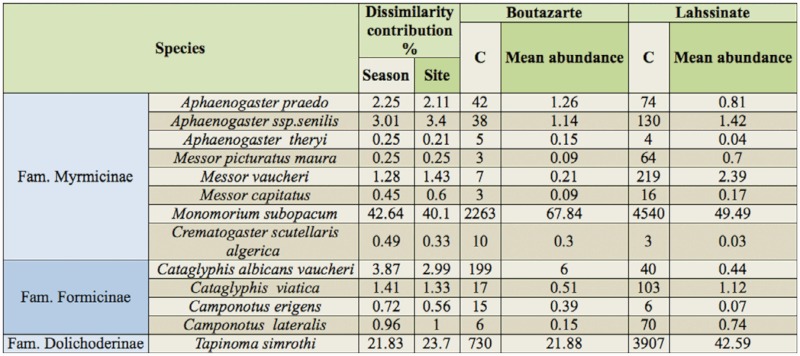
Ant species contribution to average dissimilarity (determined by SIMPER) among seasons and study sites, ant species catches (C) and mean abundance in the coastal (Boutazarte) and the semi-continental (Lahssinate) sites in the Moroccan Argan forest.

**Table 4.**
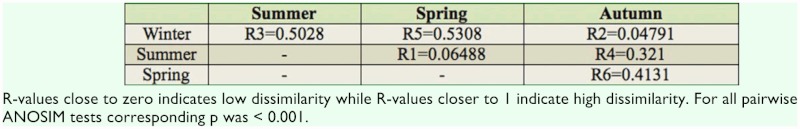
R-values of pairwise (ANOSIM, Analysis of Similarities) comparison of the ant species composition sampled during the seasons in the littoral and semi-continental sites of study at Essaouira Argan forest.

## Figures and Tables

**Appendix 1.**
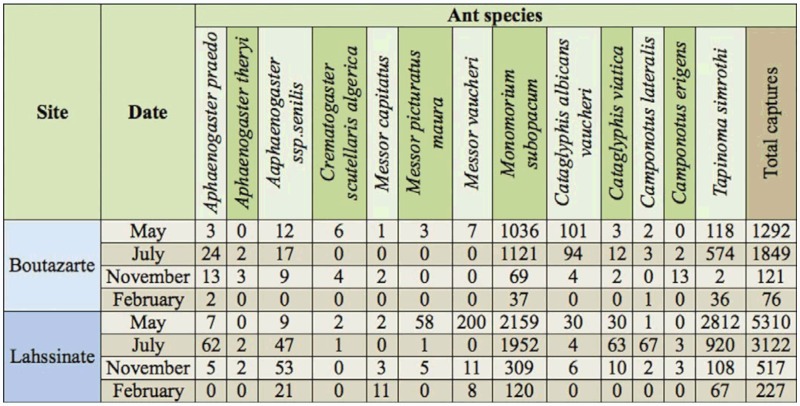
Number of workers of the different ant species caught in pitfall traps each sampling day and study site in Essaouira region (Atlantic Morocco). Boutazarte is a coastal site, and Lahssinate a semi-continental site. Pitfalls were active during 24 hours. Values are the sum of 70 pitfalls (7 pitfalls per tree × 10 trees/site).

## Abbreviation

**Ntot**,: total ant abundance
